# Virtual reality and surgical oncology

**DOI:** 10.3332/ecancer.2023.1525

**Published:** 2023-03-23

**Authors:** Peng Yun Ng, Eric G Bing, Anthony Cuevas, Ajay Aggarwal, Benjamin Chi, Sudha Sundar, Mulindi Mwanahamuntu, Miriam Mutebi, Richard Sullivan, Groesbeck P Parham

**Affiliations:** 1 King’s College London, London WC2R 2LS, UK; 2 Guy’s and St Thomas’ Trust, London SE1 9R, UK; 3 Institute for Leadership Impact, Southern Methodist University, Dallas, TX 75205, USA; 4 Department of Teaching and Learning, Technology-Enhanced Immersive Learning Cluster, Annette Simmons School of Education and Human Development, Southern Methodist University, Dallas, TX 75205, USA; 5 London School of Hygiene and Tropical Medicine, London WC1E 7HT, UK; 6 Icahn School of Medicine, New York, NY 10029-6574, USA; 7 Institute of Cancer and Genomic Sciences, University of Birmingham, Birmingham B152TT, UK; 8 Pan Birmingham Gynaecological Cancer Centre, City Hospital, Birmingham, B187QH, UK; 9 Women and Newborn Hospital, University Teaching Hospital, Lusaka 10101, Zambia; 10 Department of Surgery, Aga Khan University Hospital, Nairobi 30270-00100, Kenya; 11 Conflict & Health Research Group, King’s College London, London WC2R 2LS, UK

**Keywords:** virtual reality, augmented reality, surgical oncology, global oncology, surgery, cancer, implementation science, technology, innovation

## Abstract

More than 80% of people diagnosed with cancer will require surgery. However, less than 5% have access to safe, affordable and timely surgery in low- and middle-income countries (LMICs) settings mostly due to the lack of trained workforce. Since its creation, virtual reality (VR) has been heralded as a viable adjunct to surgical training, but its adoption in surgical oncology to date is poorly understood. We undertook a systematic review to determine the application of VR across different surgical specialties, modalities and cancer pathway globally between January 2011 and 2021. We reviewed their characteristics and respective methods of validation of 24 articles. The results revealed gaps in application and accessibility of VR with a proclivity for high-income countries and high-risk, complex oncological surgeries. There is a lack of standardisation of clinical evaluation of VR, both in terms of clinical trials and implementation science. While all VR illustrated face and content validity, only around two-third exhibited construct validity and predictive validity was lacking overall. In conclusion, the asynchrony between VR development and actual global cancer surgery demand means the technology is not effectively, efficiently and equitably utilised to realise its surgical capacity-building potential. Future research should prioritise cost-effective VR technologies with predictive validity for high demand, open cancer surgeries required in LMICs.

## Background

Of 15.2 million new patients diagnosed with cancer each year, more than 80% require surgery at least once during the course of their disease [1–3]. However, less than 5% of patients from low-income countries and around 20% of patients from middle-income countries (LMICs) have access to basic cancer surgery due to lack of human resource and inadequate healthcare infrastructure [2]. Developing novel tools as public goods to help build capacity and capability in surgical oncology across very different resource settings has become one of the most pressing global needs.

Virtual reality (VR) is defined as a computer-generated simulation of an environment, where it interacts with users in a realistic way. In the past decades, the technology has evolved from a non-immersive, two-dimensional screen-based interaction into an immersive three-dimensional experience. In an immersive VR experience, one can interact with the virtual setting in a realistic manner through digital transposition of real objects and settings. With recent technology advancement in VR which improved user orientation and user sensory experience through haptic feedback, its application gained traction not only in surgical oncology where it is heralded as an effective adjunct to surgical training, but also in different fields of cancer management from radiotherapy through to rehabilitation.

The advantages of VR simulation in surgical oncology training lie in its ability to allow trainees to learn, practice and master technical skills, clinical judgement, and the in-and-out of surgical procedures in a safe and controlled environment. This has become more important nowadays, as trainees face steeper learning curve to master increasingly complex surgical procedures with more restricted training hours and higher patient expectation. There is growing evidence behind how VR can be utilised to build surgical capacity and capability through reducing the time required of trainees to develop surgical proficiency in the operating room. Besides surgical training, VR also plays an increasingly important role in optimising operative planning through better illustration of individual anatomical structure, particularly in high-risk and complex procedures.

In non-surgical oncology, VR has also grown into a new avenue to engage with cancer patients for patient education, rehabilitation and psychosocial support. The immersive experience provides a new avenue for patients to learn the complexity of their cancer treatment, such as radiotherapy, before they consent to the treatment and experience it in reality. During and after cancer treatment, patients also engage with immersive VR experience to rehabilitate physically and to process physical and psychological stress of the treatment itself.

Given the burgeoning adoption of VR in various fields of oncology and promising early data in improving patient outcome especially for surgical oncology, an up-to-date, overarching analysis of VR technology and its application in surgical oncology will shed light on the strengths and opportunities. The primary rationale of this review is to understand how VR is being utilised in surgical oncology, situated within the wider context of VR in oncology. We identify trends across geographies, intervention, tumour type and cancer care pathway and to highlight gaps. This review also provides an in-depth analysis to identify ideal characteristics (development, validity and clinical impact) of VR surgical simulation for clinical training and to inform method validation of VR-assisted surgical tools.

## Materials and methods

We carried out a systematic review using the Web of Science following PRISMA guidance. We included papers (articles and reviews only) on VR and cancer. This process included papers published for 10 years between January 2011 and January 2021 and was done with a bibliometric filter in two parts. The first part involved the application of a pre-developed filter to identify papers on cancer research. The papers were then subsequently selected based on the application of VR if they had the words ‘virtual reality’ or ‘augmented intelligence’, or simply ‘VR’, in their titles. The full search strategy used is available in the supplementary data (Appendix A).

### Inclusion criteria

Published full-text articles describing oncology and VR were considered for inclusion. Articles must have been published in English in a peer reviewed journal between the dates specified above.

### Exclusion criteria

**Figure 1.**
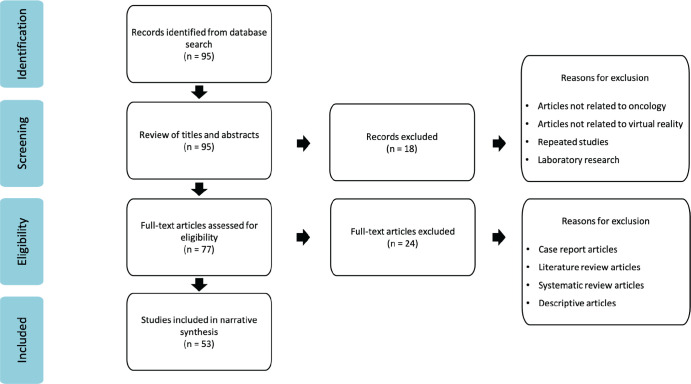
PRISMA flow chart of identification for articles for inclusion.

Any papers not related to cancer and VR were excluded (Figure 1). For example, publications describing ‘VR’ as an abbreviation of chemotherapy instead of VR were excluded. Review articles, laboratory research, case reports, conference proceedings and repeated studies were also excluded.

### Data selection

Richard Sullivan (RS) carried out the initial search. Following which, Peng Yun Ng (PN) and RS selected articles meeting inclusion criteria from titles and abstracts for full text review. PN then reviewed full text articles to consider whether inclusion and/or exclusion criteria were met. Disagreement was discussed with Ajay Aggarwal (AA) to reach a consensus.

### Data extraction

PN extracted data from included studies, with consultation from RS and AA. Data extracted from all studies included:

- location of study
- characteristics of study (funding, setting, research design and sample size)
- part of cancer care pathway where VR was applied
- purpose of VR
- modality of VR
- clinical speciality
- tumour site
- user of VR

The studies were categorised into surgical and non-surgical oncology. Studies related to surgical oncology were further compartmentalised based on the purpose of the VR, be it either for clinical training or operative planning.

For studies focusing on the use of VR for clinical training, specific data extracted included:

- characteristics of VR-assisted training tool
- method validation of VR-assisted training

The characteristics of the VR-assisted surgical training tool were set out as follows. Fidelity is defined as the extent to which the simulator describes the realism or learning experience that the simulator provides. Generally, low fidelity refers to basic psychomotor tasks while high fidelity refers to full-length operations. User feasibility is more commonly known as user acceptance rate, a measure of whether something is capable of being done or carried out. Face validity is the extent to which the examination resembles real life situations. Content validity is the extent to which the domain that is being measured is measured by the assessment tool. Construct validity is the extent to which a test measures the trait it is designed to measure; sometimes inferred as the extent to which a test discriminates between various levels of expertise. Lastly, predictive validity is the ability of examination to predict future performance of the clinician.

On the other hand, studies related to operative planning provided information on:

- pre/intra-operative use of VR-assisted tool
- surgical complication rate
- duration of procedure
- benefit of VR-assisted tool

Studies related to non-surgical oncology were divided based on the modality of the VR into radiotherapy, rehabilitation and neuropsychiatric intervention. Data were extracted for cancer speciality, purpose of intervention and outcome.

## Results

The initial search identified 95 papers. After a review of titles and abstracts, 77 articles were chosen for further review. Of these, 53 full-text articles were selected for data analysis. The study selection process followed the PRISMA guidance and is illustrated in Figure 1.

A summary of the characteristics of all [53] included studies, describing the purpose and modality of VR in the cancer care pathway is available as Appendix B.

**Figure 2.**
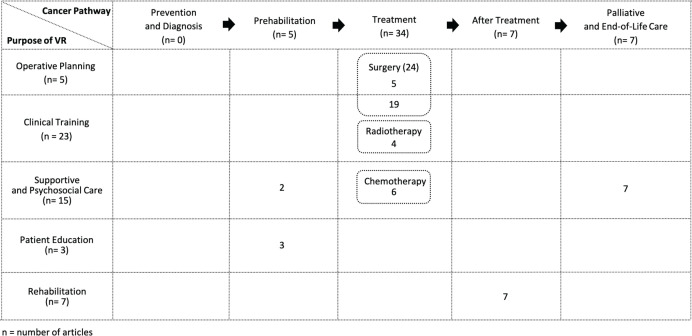
Swimlane diagram of the use of VR in the cancer care pathway.

Along the stages of the cancer care pathway, VR was applied predominantly in the delivery of cancer treatment (*n* = 34, 64.2%) with surgical oncology dominating (*n* = 24) [4–36]. Other care pathway domains where VR has been studied include post-cancer treatment supportive care (*n* = 7, 13.2%) [37–43], palliative and end-of-life care (*n* = 7, 13.2%) [44–50], and prehabilitation (*n* = 5, 9.4%) [26, 33, 44, 51–53] (Figure 2).

In terms of purpose of VR adoption, 23 studies (42.%) studied the use of VR in providing clinical training [6–12, 14–22, 24, 26–29, 32, 54]; 15 (28.3%), supportive and psychosocial care related to cancer [4, 23, 31, 34–36, 44–50, 55, 56]; 7 (13.2%), rehabilitation [37–43]; 5 (9.4%), operative planning [5, 13, 25, 30, 33], and 3 (5.7%), patient education [51–53].

**Figure 3.**
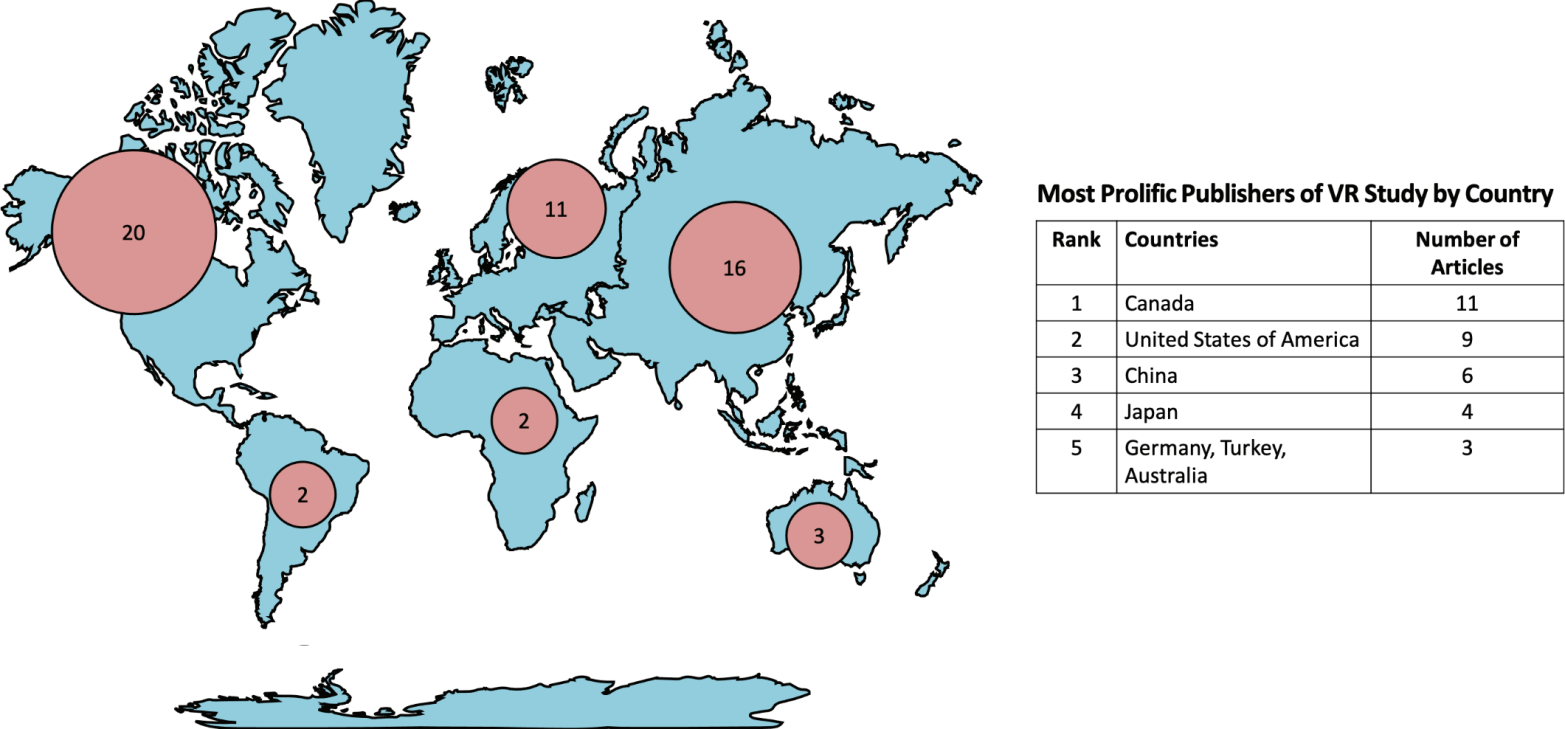
Number of VR studies related to oncology by continent and country.

The majority of VR studies in oncology were from North America continent (*n* = 20, 37.8%) [4, 6, 8, 10–12, 16, 17, 25, 28, 33, 34, 37, 50, 51, 53–56]. Asia (*n* = 16, 30.2%) is the second most prolific continent [5, 7, 9, 13, 18, 20, 21, 29, 35, 36, 38, 39, 41, 43, 44, 47], followed by Europe (*n* = 11, 20.8%) [15, 19, 22, 24, 31, 32, 45, 46, 48]. Fewer articles were published from centres based in Australia (*n* = 3, 5.7%), [27, 49, 52] Africa (*n* = 2, 3.8%), [23, 26] and South America (*n* = 2, 3.8%) [20, 40]. This geographical distribution is visualised as hotspots on a world map in Figure 3. Canada (*n* = 11, 20.8%), [8, 11, 12, 14, 16, 17, 28, 50, 53–55] United States of America (*n* = 9, 17.0%) [4, 6, 10, 25, 33, 34, 37, 51, 56], and China (*n* = 6, 11.3%) [5, 7, 13, 18, 29, 44] in consecutive order, are the top three countries for VR research in oncology.

**Figure 4.**
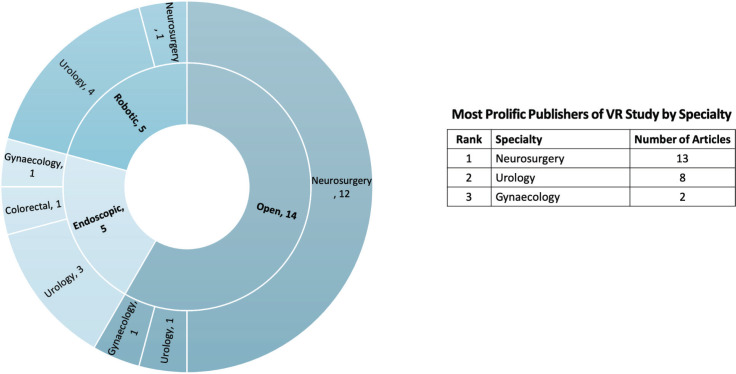
Number of VR studies by surgical modality and specialty.

Of the 24 studies related to surgical oncology, 19 (79.2%) focused on the use of VR in supporting surgical trainees with competencies, [7–12, 14–17, 19, 20, 54] and the other five (20.8%) focused on pre-operative surgical planning [5, 13, 25, 30, 33]. VR was applied across different modalities of surgery, including open procedures (*n* = 14, 58.3%), [5, 8, 11–14, 16, 17, 26, 28–30, 33, 54], minimally invasive (*n* = 5, 20.8%) [7, 9, 10, 19, 22], and robotic (*n* = 5, 20.8%) procedures [15, 20, 21, 25, 32]. Neurosurgical oncology procedures were the most studied in terms of VR application (*n* = 13, 54.2%), followed by urology (*n* = 8, 33.3%), and gynaecology (*n* = 2, 8.3%), as illustrated in Figure 4.

In terms of characteristics of VR-assisted surgical training tools, 11 (57.9%) out of the 19 articles described VR-assisted tools which have high fidelity, meaning clinicians were able to practice through simulation of full length operations instead of abstract psychomotor tasks [7–10, 12, 15, 17, 19, 22, 26, 32]. 12 (63.2%) out of 19 articles further identified VR-assisted surgical training tools paired with haptic feedback to enhance the realism of the simulation to operation [8, 10, 11, 14, 16, 17, 19, 20, 22, 28, 54]. As such, clinicians received sensory feedback using transmitted resistance when they encountered tissues or objects during the simulation.

While all the VR-assisted surgical tool illustrated face and content validity, only 12 (63.2%) exhibited construct validity, thus discriminating clinicians of various levels of expertise [11, 12, 14–17, 21, 22, 26, 32, 54]. Only one (5.3%) study demonstrated predictive validity, where the VR-assisted training was shown to improve clinicians’ future performance [21]. However, the study only has a sample size of three, undermining the internal and external validity of its result.

In addition to the characteristics of VR-assisted surgical training tool shown in Table 1, a range of methods used to validate VR-assisted surgical training were consolidated in Table 2. Surgical competency was mostly validated using dexterity parameters (*n* =17, 89.5%) [8–12, 14–17, 20–22, 26, 28, 32, 54] and objective structured assessment of technical skills (OSATS) (*n* = 14, 73.7%) [7–12, 15, 16, 19–22, 26, 32, 54]. Psychomotor tasks were used in 11 out of 19 of the studies (57.9%) [7, 8, 10, 11, 14–17, 21, 26, 32]. One study (5.3%) used knowledge-based validation instead [29].

14 out of 19 studies (73.7%) recorded the time taken to complete the task, [7–12, 15, 17, 19, 21, 22, 26, 32] while 9 (47.4%) and 8 (42.1%) included post-operative complications, specifically tissue injury [7, 8, 11, 12, 15, 19, 22, 32, 54] and bleeding [7, 8, 11, 12, 19, 22, 32, 54] respectively, as part of the training validation. Only five (26.3%) studies assessed the subjective competence of clinicians using the Likert Scale [8, 19, 22, 26, 29]. No studies demonstrated test-retest reliability in its validation process or assessed for inter-rater reliability.

The application of VR in surgical oncology was not limited to surgical training, but also operative optimisation in clinical setting. Five out of 24 studies related to surgical oncology highlighted the use of VR in operative planning. Of the five studies, three (60%) were used in pre-operative setting [5, 30, 33] and two (40%) in intra-operative setting [13, 25]. It is worth noting that only one study (20%) reported an improvement in surgical outcome in terms of resection rate and the preservation of neural function [13]. The other four (80%) highlighted its use in providing anatomic information [5, 25, 30, 33] and of these, two (40%) used the information to advise surgical approach [5, 30]. A summary table for these five studies is attached as Appendix C.

## Discussion

From a nascent technology reserved for experts from niche computer field, VR tool has matured and evolved into a popular technological gadget in the general consumer market as its creation fundamentally changes how one engages with virtuality for entertainment, education and communication.

In particular, VR in medicine has emerged as an epiphenomenon of the rapid pace of its technological advancement as its potential was widely regarded as undertapped since an early stage. Nonetheless, our comprehensive search discovered surprisingly little literature related to its application in surgical oncology and, more widely, oncology. The literature reflects a widely disparate range of applications at different stages of cancer pathway and for different purposes, such as providing supportive and psychosocial care and facilitating rehabilitation. However, surgical oncology remains the main focus both for surgical training and operative planning.

### Application gap between high-risk, complex and low-risk, open procedures

As a surgical oncology speciality, neuro-oncology led in the research and adoption of VR, primarily in surgical training. Most studies highlighted the benefit of VR simulation for neurosurgery trainees in gaining skill-based experience in common yet high-risk neurosurgeries, which are often heavily led by consultants in the operating theatre. Trainees were able to practice and receive feedback from senior colleagues on a wide range of surgical skills using VR simulation, without detriment to patients and with hope to translate acquired simulation-based skills to surgical proficiency. Their competencies were often subsequently tested through psychomotor tasks or OSATS to consolidate learning.

Besides the high risk attribute of procedure, technically complex surgeries, such as those requiring endoscopic and robotic equipment, are also bona fide early adopters of VR simulation in surgical training. This is due to the fact that surgeons generally require more time and practice to acquire new skillsets required to use endoscopy or robot, which they often initially lack human intuition in without prior clinical exposure and experience. This review identified uro-oncology as the leading surgical specialty on this front, driven primarily by novel endoscopic and robotic approach to prostatectomy.

This pattern of adoption of VR overshadows the larger potential of VR simulation as a platform to develop surgical training towards high demand, low-risk, open-approach cancer surgeries such as mastectomy and colectomy, that are sorely lacking in capacity in LMICs.

### Accessibility gap between high-income and LMICs

There were very few studies of VR conducted by and for LMICs (except for China). This reflects a yawning socio-techncological gap between the direction of travel of VR in surgical oncology and the actual development need of VR to build surgical capacity and capability globally.

Nonetheless, one study from Zambia [26] stood out as an anomaly, as it trialled the use of a cost-efficient VR (Oculus Rift, each cost less than USD1,500) as a surgical training tool for trainees to learn open radical abdominal hysterectomy for cervical cancer in local setting. It demonstrated how the VR simulation, when co-designed and co-developed with local experts, can teach surgical novices and may lead to shorter surgical training time and better surgical outcome. Appendix D attached includes two images, depicting the VR of an operating theatre mimicking the real setting of a Zambian teaching hospital and that of an intricate step of an open abdominal hysterectomy using equipment available locally. This example sheds optimism on the potential of VR as the scalable and sustainable solution to addressing global surgical training needs in oncology when its design fits the demand. To replicate such practice, more publically funded development and implementation of VR are necessary to drive its adoption. Otherwise, the tendency for VR to service solely high-income countries and/or technically complex procedures (MIS/robotics) is likely to prevail.

### Evaluation and method validation gap in VR-assisted surgical training

Given the relative novelty of VR as a surgical training tool, there remains a dearth of evidence that actually showed VR simulation improves cancer surgical training, surgical quality and better patient outcomes. Evaluation gap is defined by the lack of standardisation of ideal qualities in VR-assisted surgical training tool, which make them user-friendly, realistic and effective in developing surgical skills. Based on definition set by Lewis *et al* [57], most VR in studies have attained face and content validity with the adoption of high-fidelity simulation, advanced VR lens for orientation and haptic feedback. However, many still struggle to achieve contsruct and predictive validity, which enable surgical trainees to track their progress at different level of proficiency and to measure translation of their acquired surgical proficiency into better surgical care and patient outcomes based on real-life performance, respectively.

Such evaluation gap is thought to be tightly correlated to a lack of gold standard to validate VR-assisted surgical training tools, also known as method validation gap. This review identified heterogenous methods adopted to validate surgical training, most commonly through psychomotor tasks and OSATS that measure dexterity parameters, time taken to complete task and/or complication rate. Such practice is often enabled by the inbuilt features of the simulators but often lacks in inter-rater and test-retest reliability, which are crucial in distinguishing the level of proficiency of trainee through a peer-review process and in testing their future operative performance.

Thus, to bridge these gaps, we recommend future studies related to VR-assisted surgical training to include a validation process, which requires more than one senior reviewer and to follow up on trainees’ surgical proficiency as well as patient outcome to establish test-retest reliability and predictive validity, respectively.

### VR in non-surgical oncology

Outside the scope of surgical oncology, the application of VR emerges in a myriad of other oncology fields- most prominently radiotherapy, neuropsychiatry and rehabilitation. These three modalities of treatment share the common feature of utilising the fully immersive experience of VR to engage with patients meaningfully for an extended period of time. To illustrate, half (three out of six) of the radiotherapy-related studies demonstrated how VR was adopted to help patients comprehend the intricate process of radiotherapy and vivaciously experience the treatment setting prior to starting, thus easing anxiety. On the other hand, 13 out of 15 studies which used VR as a neuropsychiatric intervention showed how VR could be effective in easing pain through distraction and alleviate patient mood. Creatively, mainstream VR consoles, such as Xbox 360 and Wii, have also over time adapted their games to help patients rehabilitate limb function and improve energy level after cancer treatment.

## Conclusion

The emergence of VR in medicine opens a window of opportunity to build capacity and capability in surgical oncology in a wide range of resource settings using low cost novel tools. However, current VR development is still overly focused on high-income settings, with complex procedures such as MIS and robotics, instead of open-approach procedures needed in many LMIC settings. The asynchrony between the development of VR in surgical oncology and the actual global cancer surgery demand means that the technology is not effectively, efficiently and equitably applied to realise its capacity-building potential. Future research should prioritise the use of affordable VR technology in high-demand surgical procedures and further explore its use in other common cancer domains such as breast, colorectal and lung cancer. This process can be accelerated with the adoption of a more standardised and complete method validation of VR-assisted surgical training tool, in a bid to make VR a real and effective digital adjunct to traditional, didactic surgical training.

## Conflicts of interest

N/A.

## Authors’ contributions

PN, RS and AA conceived, designed, and supervised the study. PN and RS searched, screened, and assessed the publications. PN extracted, analysed and visualised the data. PN and RS drafted the manuscript and interpreted the findings. EB, RS, AA, AC, BC, MM, MM and GP reviewed and edited drafts of the manuscript. RS accessed and verified the data. All authors read the manuscript, provided feedback, and approved the final version.

## Figures and Tables

**Table 1.** Characteristics of VR-assisted surgical training tool.

Reference year/location	Author	Simulator	Specialty	Fidelity	Haptic feedback	User feasibility	Face validity	Content validity	Construct validity	Predictive validity
2013, China	Zhu *et al* [7]	TURPSim™	Urology (TURP)	High	No	Yes	Yes	Yes	No	No
2014, Canada	Gelinas-Phaneuf *et al* [8]	Neurotouch	Neurosurgery (Meningioma)	High	Yes	Yes	Yes	Yes	No	No
2014, Turkey	Akdemir *et al* [9]	LapSim Gynae	Gynaecology (Bilateral Tubal Ligation)	High	No	Yes	Yes	Yes	No	No
2014, USA	White *et al* [10]	Accutouch	Colorectal (Endoscopy)	High	Yes	Yes	Yes	Yes	No	No
2015, Canada	Alotaibi *et al* [54]	Neurotouch	Neurosurgery (Meningioma)	Low	Yes	Yes	Yes	Yes	Yes	No
2015, Canada	Azarnoush *et al* [11]	Neurotouch	Neurosurgery (Meningioma)	Low	Yes	Yes	Yes	Yes	Yes	No
2015, Canada	Alzhrani *et al* [12]	Neurotouch	Neurosurgery (Meningioma)	High	Yes	Yes	Yes	Yes	Yes	No
2017, Canada	Azarnoush *et al* [14]	NeuroVR	Neurosurgery (Meningioma)	Low	Yes	Yes	Yes	Yes	Yes	No
2018, UK	Harrison *et al* [15]	RobotiX Mentor	Urology (Radical Prostatectomy)	High	No	Yes	Yes	Yes	Yes	No
2018 Canada	Sawaya *et al* [16]	NeuroVR	Neurosurgery (Intracranial Tumour)	High	Yes	Yes	Yes	Yes	Yes	No
2018, Canada	Sawaya *et al* [17]	NeuroVR	Neurosurgery (Meningioma)	Low	Yes	Yes	Yes	Yes	Yes	No
2018, Germany	Neumann *et al* [19]	Uro-Trainer	Urology (Cystoscopy, TURBT)	High	Yes	Yes	Yes	Yes	No	No
2018, Japan, Mexico	Heredia-Perez *et al* [20]	MM-2 Robot	Neurosurgery (Transpenoidal Pituitary Tumour)	Low	Yes	Yes	Yes	Yes	No	No
2018, Korea	Shim *et al* [21]	da Vinci Skills Simulator	Urology (VUA of RARP)	Low	No	Yes	Yes	Yes	Yes	Yes
2019, Germany	Schulz *et al* [22]	Uro-Trainer	Urology (TURBT)	High	Yes	Yes	Yes	Yes	Yes	No
2019, Zambia	Bing *et al* [26]	Oculus Rift	Gynaecology (Total Abdominal Hysterectomy)	High	No	Yes	Yes	Yes	Yes	No
2020, Canada	Siyar *et al* [28]	NeuroVR	Neurosurgery (Intracranial Tumour)	Low	Yes	Yes	Yes	Yes	Yes	No
2020, China	Shao *et al* [29]	Microsoft Hololens®	Neurosurgery (Skull-based tumour)	Low	No	Yes	Yes	Yes	No	No
2020, Sweden	Ebbing *et al* [32]	RobotiX Mentor	Urology (Non-guided bladder neck dissection and neurovascular bundle dissection)	High	No	Yes	Yes	Yes	Yes	No

**Table 2.** Method validation of VR-assisted surgical training.

Reference year/location	Author	Simulator	Psychomotor task		Knowledge-based validation	Dexterity parameters	Time taken to complete	Tissue injury	Bleeding	Subjective competence	Inter-rater reliability	Test-retest reliability
2013, China	Zhu *et al* [7]	TURPSim™	Yes	Yes	No	No	Yes	Yes	Yes	No	No	No
2014, Canada	Gelinas-Phaneuf *et al* [8]	Neurotouch	Yes	Yes	No	Yes	Yes	Yes	Yes	Yes	No	No
2014, Turkey	Akdemir *et al* [9]	LapSim Gynae	No	Yes	No	Yes	Yes	No	No	No	No	No
2014, USA	White *et al* [10]	AccuTouch	Yes	Yes	No	Yes	Yes	No	No	No	No	No
2015, Canada	Alotaibi *et al* [54]	Neurotouch	No	Yes	No	Yes	Yes	Yes	Yes	No	No	No
2015, Canada	Azarnoush *et al* [11]	Neurotouch	Yes	Yes	No	Yes	Yes	Yes	Yes	No	No	No
2015, Canada	Alzhrani *et al* [12]	Neurotouch	No	Yes	No	Yes	Yes	Yes	Yes	No	No	No
2017, Canada	Azarnoush *et al* [14]	Neurotouch	Yes	No	No	Yes	No	No	No	No	No	No
2018, UK	Harrison *et al* [15]	RobotiX Mentor	Yes	Yes	No	Yes	Yes	Yes	No	No	No	No
2018 Canada	Sawaya *et al* [16]	NeuroVR	Yes	Yes	No	Yes	Yes	No	No	No	No	No
2018, Canada	Sawaya *et al* [17]	NeuroVR	Yes	No	No	Yes	No	No	No	No	No	No
2018, Germany	Neumann *et al* [19]	Uro-Trainer	No	Yes	No	Yes	Yes	Yes	Yes	No	No	No
2018, Japan, Mexico	Heredia-Perez *et al* [20]	MM-2 Robot	No	Yes	No	Yes	No	No	No	No	No	No
2018, Korea	Shim *et al* [21]	da Vinci Skills Simulator	Yes	Yes	No	Yes	Yes	No	No	No	No	No
2019, Germany	Schulz *et al* [22]	Uro-Trainer	No	Yes	No	Yes	Yes	Yes	Yes	Yes	No	No
2019, Zambia	Bing *et al* [26]	Oculus Rift	Yes	Yes	No	Yes	Yes	No	No	Yes	No	No
2020, Canada	Siyar *et al* [28]	NeuroVR	No	No	No	Yes	No	No	No	No	No	No
2020, China	Shao *et al* [29]	Microsoft Hololens®	No	No	Yes	No	No	No	No	Yes	No	No
2020, Sweden	Ebbing *et al* [32]	RobotiX Mentor	Yes	Yes	No	Yes	Yes	Yes	Yes	Yes	no	No

## Supplementary information

**Figshare Link:** https://doi.org/10.6084/m9.figshare.22207594.v1
